# Acoustic and Natural Language Markers for Bipolar Disorder: A Pilot, mHealth Cross-Sectional Study

**DOI:** 10.2196/65555

**Published:** 2025-04-16

**Authors:** Cristina Crocamo, Riccardo Matteo Cioni, Aurelia Canestro, Christian Nasti, Dario Palpella, Susanna Piacenti, Alessandra Bartoccetti, Martina Re, Valentina Simonetti, Chiara Barattieri di San Pietro, Maria Bulgheroni, Francesco Bartoli, Giuseppe Carrà

**Affiliations:** 1 School of Medicine and Surgery, University of Milano-Bicocca, via Cadore 48, Monza, 20900, Italy, 39 0264488483; 2 Ab.Acus, Milan, Italy; 3 Laboratory of Neurolinguistics and Experimental Pragmatics (NEP), University School for Advanced Studies IUSS, Pavia, Italy

**Keywords:** digital mental health, remote assessment, mHealth, speech, NLP, natural language processing, acoustic, symptom severity, machine learning, markers, mental health, bipolar disorders, app, applications, multimodal, mobile health, voice, vocal, bipolar, verbal, emotion, emotional, psychiatry, psychiatric, mental illness

## Abstract

**Background:**

Monitoring symptoms of bipolar disorder (BD) is a challenge faced by mental health services. Speech patterns are crucial in assessing the current experiences, emotions, and thought patterns of people with BD. Natural language processing (NLP) and acoustic signal processing may support ongoing BD assessment within a mobile health (mHealth) framework.

**Objective:**

Using both acoustic and NLP-based features from the speech of people with BD, we built an app-based tool and tested its feasibility and performance to remotely assess the individual clinical status.

**Methods:**

We carried out a pilot, observational study, sampling adults diagnosed with BD from the caseload of the Nord Milano Mental Health Trust (Italy) to explore the relationship between selected speech features and symptom severity and to test their potential to remotely assess mental health status. Symptom severity assessment was based on clinician ratings, using the Young Mania Rating Scale (YMRS) and Montgomery-Åsberg Depression Rating Scale (MADRS) for manic and depressive symptoms, respectively. Leveraging a digital health tool embedded in a mobile app, which records and processes speech, participants self-administered verbal performance tasks. Both NLP-based and acoustic features were extracted, testing associations with mood states and exploiting machine learning approaches based on random forest models.

**Results:**

We included 32 subjects (mean [SD] age 49.6 [14.3] years; 50% [16/32] females) with a MADRS median (IQR) score of 13 (21) and a YMRS median (IQR) score of 5 (16). Participants freely managed the digital environment of the app, without perceiving it as intrusive and reporting an acceptable system usability level (average score 73.5, SD 19.7). Small-to-moderate correlations between speech features and symptom severity were uncovered, with sex-based differences in predictive capability. Higher latency time (*ρ*=0.152), increased silences (*ρ*=0.416), and vocal perturbations correlated with depressive symptomatology. Pressure of speech based on the mean intraword time (*ρ*=–0.343) and lower voice instability based on jitter-related parameters (*ρ* ranging from –0.19 to –0.27) were detected for manic symptoms. However, a higher contribution of NLP-based and conversational features, rather than acoustic features, was uncovered, especially for predictive models for depressive symptom severity (NLP-based: *R*^2^=0.25, mean squared error [MSE]=110.07, mean absolute error [MAE]=8.17; acoustics: *R*^2^=0.11, MSE=133.75, MAE=8.86; combined: *R*^2^=0.16; MSE=118.53, MAE=8.68).

**Conclusions:**

Remotely collected speech patterns, including both linguistic and acoustic features, are associated with symptom severity levels and may help differentiate clinical conditions in individuals with BD during their mood state assessments. In the future, multimodal, smartphone-integrated digital ecological momentary assessments could serve as a powerful tool for clinical purposes, remotely complementing standard, in-person mental health evaluations.

## Introduction

Bipolar disorder (BD) is a lifelong, episodic illness characterized by mood recurrences, including manic or hypomanic, depressive, and mixed episodes [1-3]. The burden associated with BD, affecting families, carers, and mental health care systems, is heavy [4]. Community services often struggle in delivering regular monitoring of BD treatment needs, resulting in relapses that seem difficult to predict [4-6].

Language disturbances are among the core symptoms of acute episodes in BD, since speech patterns are modulated by the emotional and neurophysiological status [7,8]. Therefore, language may play a key role in the assessment of an individual’s current experiences, emotions, thought patterns, and symptoms. While content analysis may reveal grandiosity associated with elevated mood, impulsivity, or changes in goal-directed activities, natural language may provide insights into mood fluctuations, cognitive processes, and behavioral patterns [9]. In particular, changes in the rate of speech are likely to indicate mood oscillations, including pressure of speech and increased verbosity during manic episodes [10] and poverty of speech and increased pause times during depressive episodes [11-13]. Clinicians are trained to recognize variations in language and voice, along with gestures and facial expressions, implicitly assessing both coherence and organization of speech and natural language features. However, this process is inevitably vulnerable to inconsistencies and biases.

Recent research in mental health and computer science has put forward computational approaches for speech analysis across a variety of mental disorders, proposing automated methods to assess and monitor the individual’s mental state through speech patterns [14-18]. Promising techniques in speech acoustic signal processing [10,11,17,19-21], using mobile health (mHealth) technology, can bridge subjective and objective components across various stages, such as prediction of illness onset, diagnostic processes, assessment of severity, and forecast of treatment outcomes [22-25]. Indeed, natural language processing (NLP) techniques, exploring language resources (eg, lexical choices, syntax, and semantics) both qualitatively and quantitatively (eg, topic modeling, clustering, and classification), may produce deeper insights across different clinical conditions [9,26]. For example, observable linguistic traits (eg, increased use of both first-person pronouns and negative emotion expressions) can be identified among people with BD [23]. However, although linguistic features are informative, they are context-dependent and inferred according to word transcriptions [27]. Thus, speech analyses combining acoustic-dependent features (eg, speech prosody and voice quality) with NLP-based measures appear more promising in terms of model predictions, possibly providing a more accurate mental health assessment [23,27,28].

Indeed, research has shown that acoustic features are markers of emotional states in BD [29], and that quantifiable speech differences can predict the scores of scales such as the Young Mania Rating Scale (YMRS) and the Montgomery-Åsberg Depression Rating Scale (MADRS) [13,27]. On the other hand, recent evidence has shown how smartphone-based voice data [30] can enhance BD monitoring in real time, detecting possible mood changes [31,32]. Thus, speech-based systems embedded in smartphones might be useful tools for complementary, continuous assessments of BD clinical states. We therefore built an app-based tool, jointly using acoustic and NLP-based features from the speech of people with BD who delivered a narrative, and carried out a pilot study aimed at testing its feasibility and performance to remotely assess the individual clinical status. Continuous, uninterrupted spoken accounts, as supplied by individuals, provided the unique opportunity to combine communication style information from an in-depth set of acoustic features and NLP-based scores as potential digital markers of symptom severity in speech. We rigorously chose to test the tool’s performance against standard psychometric assessments of mania and depression in order to explore its potential for remote, complementary assessments.

## Methods

The report of this study adheres to the STROBE (Strengthening the Reporting of Observational Studies in Epidemiology) statement (checklist presented in Multimedia Appendix 1) [33].

### Study Design and Sampling Strategies

We conducted a pilot, cross-sectional study involving adult participants (aged 18 years or older) from the caseload of the Nord Milano Mental Health Trust (Italy). The Trust includes 2 psychiatric intensive care units, with a total of 27 beds, and also provides community mental health care for the same 280,000 inhabitants of the northern area of the Metropolitan City of Milan through 4 community mental health teams with multidisciplinary staff. The relevant catchment area comprises highly urbanized, both deprived and affluent, districts.

Inclusion criteria comprised a diagnosis of BD and the willingness to participate in the study. People with physical impairments affecting their acoustic capabilities were excluded. Based on inclusion and exclusion criteria, eligible individuals were identified among individuals consecutively admitted to the Trust. Then, they were approached by the research team, explaining the purpose of the study and, if any, potential risks.

### Ethical Considerations

Recruitment efforts were carried out in accordance with ethical guidelines to ensure the well-being and safety of all participants. Study participants signed a written informed consent and were not compensated for their involvement. The study received ethical approval (protocol number 172‐17032023) from the local ethical committee. To maintain participant privacy and confidentiality, all study data were pseudonymized prior to analysis. No individual participants are identifiable in any images included in this manuscript or Multimedia Appendices.

### Procedures

Acoustic data were retrieved by asking participants to self-administer verbal performance tasks through a mobile app on their smartphones (SPEAKapp; [34]). Clinical testing and app usage took place on the same day in the study setting (inpatient and outpatient services). Then, the System Usability Scale (SUS), a short 10-item questionnaire based on a 5-point Likert scale, was administered to assess the usability [35] of the app.

Verbal performance in terms of prose recall was based on the Babcock test [36], for which participants were asked to listen to a short story characterized by graphic and intense contents (eg, a death in a car crash) and then to repeat what she or he remembered from this narrative. This enabled to capture speech timing patterns based on sustained speech samples.

The app gathered participants’ verbal production by using the smartphone-integrated microphone, recording and processing participants’ speech by leveraging Google Speech-To-Text APIs [37] and Python libraries (eg, Parselmouth for the Praat software [38]). Recordings involved the use of one audio channel based on the participant’s voice in a controlled environment with minimal acoustic conditions. Both the raw audio data and the transcribed text content were processed to extract acoustic and NLP-based features from speech outputs. NLP and acoustic signal models were embedded in the backend part of the mobile app.

### Measures

Consistent with recent evidence, we assumed speech as verbal behavior, the spoken output of the mental system underlying the language [39]. Through speech recognition, acoustic and linguistic features were extracted. Then, based on both NLP and acoustic features, we considered a multidimensional framework in order to generate appropriate discriminative information for the potential use of speech patterns as digital markers in BD [27,31]. A full description of selected features is provided in Table S1 in Multimedia Appendix 2.

### NLP-Based, Semantic, and Conversational Indices

NLP-based scores were computed according to distributional semantic models, encompassing vectorial representations for the meaning of words in a multi-dimensional space.

Standard linguistic scores included both the number of words, indicative of poverty of speech, and the number of words produced that matched the story text. On the other hand, novel NLP-based scores integrated mean intraword time, estimating the average time taken to articulate or pronounce subsequent words, as an indicator of processing speed, as well as word mover’s distance (WMD), capturing both lexical overlap and semantic similarity. In particular, WMD was estimated as the minimum cumulative distance between words required to exactly match the point cloud of the text of the full correct story (ie, the content distance between the full correct story and the story narrative produced by the participant), thus incorporating the semantic similarity between individual word pairs into the word distance metric [40]. In addition, latency time was calculated as a novel NLP-based score, taking into account the delay between the initiation of a spoken utterance or action and the production of its intended outcome or response when starting the task (ie, the first word).

Additional objective information was extracted from speech data. These quantitative measures included (1) speech duration, (2) speaking time (ie, phonation), (3) silence, (4) ratios of speaking time to speech duration as well as of silence to speaking time, and (5) speech rate.

### Acoustic Indices From Vocal Signals (Prosodic Cues Indices)

Measures for prosodic cues (acoustic indices quantifying how people talk during conversations) were based on the signal’s frequency and energy or amplitude. These were assumed to contribute to conveying paralinguistic meaning [41]. Based on nontextual data, acoustic components of speech were defined as the key phonetic elements, that is, objectively and reproducibly quantified speech sounds [27,42]. Fundamental frequency (F0) was measured by the frequency of phonation [43]. The short-term instability of the vibration of the vocal cords during phonation (ie, jitter-related indices) was also extracted (Table S1 in Multimedia Appendix 2). Higher jitter values indicated speech patterns likely characterized by irregularities or hesitations, thus mirroring potential underlying psychological distress or emotional instability. Furthermore, microperturbations of the ampleness of the signal (ie, how variable acoustic peaks refer to the period-to-period variability of the signal peak-to-peak amplitude) were identified as small fluctuations in the intensity of vocal sound waves by shimmer-related measures, with higher values indicating greater variability or instability, while lower ones suggesting more stable vocal intensity (ie, smoother and more regular speech production).

Since both periodic and nonperiodic sound waves may characterize the voice, the mean harmonics-to-noise ratio was used to measure the relationship between harmonic and nonharmonic voice elements. Noisier, more raucous voices (ie, not smooth or clear) were expected to show lower harmonics-to-noise ratios, indicating vocal cord tension or irritation, possibly suggesting emotional distress.

### Psychometric Measures

Diagnosis of BD was confirmed by the Structured Clinical Interview for *DSM-5* (*Diagnostic and Statistical Manual of Mental Disorders, Fifth Edition*; SCID-5). Based on clinician-rated assessments, depressive symptom severity was measured by the MADRS [44], while YMRS was used to assess manic symptoms [45]. Scores ranged from 0 to 60 for both MADRS [44] and YMRS [45]. In addition, cutoffs for severe mood symptoms were either a YMRS score ≥20 [46,47] or a MADRS score ≥19 [48].

### Statistical Analyses

First, we summarized participants’ characteristics, providing standard statistics for continuous and categorical variables. For both MADRS and YMRS, continuous scores were used. However, a supplementary analysis was performed based on clinically meaningful thresholds for symptom severity. A bivariate analysis was then carried out to measure the strength of the potential association between speech indices and psychometric measures. Features’ summary statistics were plotted, and correlation coefficients (Pearson and Spearman, according to assumptions on data distribution, eg, normality) were estimated. Color gradient heat plots were also generated for data visualization. Taking into account potential sex differences in speech acoustic indices [49-51], subgroup analyses were performed. Statistical significance was set at *P*<.05.

Second, based on state-of-the-art algorithms, NLP and acoustic features extracted from natural language and audio streams (Table S1 in Multimedia Appendix 2) were used to train machine-learning models to detect depressive and manic states by means of scores from MADRS and YMRS. Data were randomly split using a 5-fold nested cross-validation approach for training and testing in order to provide an unbiased evaluation of the model’s performance. In particular, random forest (RF) models, with the potential to handle both linear and nonlinear relationships between features and the target variable, were implemented. The supervised learning algorithm, with no assumptions about the distribution of the target variable, was based on the ensemble learning method of different decision trees, whose predictions were aggregated using the scikit-learn library in Python. Exploiting the bagging techniques, building multiple decision trees, RF contributed to minimizing overfitting issues by randomizing the feature selection during each tree split. This was assumed to reduce sensitivity to noise and to make decision trees less correlated through the use of a unique subset of the initial data for every base model. Moreover, we deemed features scaling unnecessary due to both the properties of the RF model and the performance metrics of comparisons. Relevant models were trained to test final performance by metrics (ie, mean squared error [MSE], mean absolute error [MAE], and *R*-squared [*R*^2^]). These tested overall performance, even controlling for sex. Shapley Additive Explanations values, showing features’ impact, were plotted. Data were analyzed using Stata release 18 and Python (version 3.10.9).

## Results

### Sample Characteristics

We included 32 subjects with BD (mean age 49.6, SD 14.3 years; 50% [16/32] females). The mean (SD) age at onset was 24.4 (10) years. As a whole, participants experienced more manic (median 4, IQR 8) than depressive episodes (median 2, IQR 5). About 40% (12/32) of participants reported a previous mood episode within 1 year before study enrollment. The MADRS median (IQR) score was 13 (21), while the YMRS median (IQR) score was 5 (16). Considering the app usage, participants reported high SUS scores on average (mean 73.5, SD 19.7). Demographic and clinical details are fully provided in Table 1.

**Table 1.** Sample^a^ characteristics.

Characteristics	BD^b^ (N=32)
Sex, n (%)	
Female	16 (50)
Male	16 (50)
Age (years), mean (SD)	49.6 (14.3)
Marital status, n (%)	
In a relationship	12 (37)
Family situation, n (%)	
Living alone	11 (34)
Education, n (%)^c^	
Elementary	1 (3)
Middle	12 (37)
High	13 (41)
University or superior	5 (16)
Employment, n (%)^c^	
Employed	13 (41)
Setting, n (%)	
Outpatient	13 (41)
Inpatient	19 (59)
Polarity of first episode, n (%)^c^	
Depressive	12 (38)
Hypomaniac or maniac	13 (41)
Unknown	1 (3)
Age of onset^c^ (years), mean (SD)	24.4 (10)
Family history^c^	11 (34%)
Hospitalizations, median (IQR)	
Lifetime	3 (7.5)
12 months	1 (2)
Suicide attempts (lifetime), n (%)	10 (31)
Alcohol use disorder (lifetime), n (%)	3 (9)
Substance use disorder (lifetime), n (%)	6 (19)
Medication, n (%)	
FGA^d^	6 (19)
SGA^e^	28 (87)
Mood stabilizer	26 (81)
Antidepressant	8 (25)
Benzodiazepine	16 (50)
Psychometric assessment	
Depressive symptoms (MADRS^f^), median (IQR)	13 (21)
MADRS <19, n (%)	17 (53)
MADRS ≥19, n (%)	15 (47)
Manic symptoms (YMRS^g^), median (SD)	5 (16)
YMRS <20, n (%)	26 (81)
YMRS ≥20, n (%)	6 (19)
SUS^h^ score, mean (SD)	73.5 (19.7)

a The sample is for a pilot, cross-sectional study in Italy.

b BD: bipolar disorder.

c Missing values: education (1), employment (2), age of onset (10), polarity of first episode (6), family history (10), alcohol use disorder (2), substance use disorder (1), FGA (2), SGA (1), mood stabilizer (2), antidepressant (3), benzodiazepine (4).

d FGA: first-generation antipsychotics.

e SGA: second-generation antipsychotics.

f MADRS: Montgomery-Åsberg Depression Rating Scale.

g YMRS: Young Mania Rating Scale.

h SUS: System Usability Scale (range 0‐100).

### Associations Between Symptom Severity and Speech Features

For descriptive purposes, NLP-based, conversational, and acoustic features are summarized in Figures S1A-S1D and S2A-S2D in Multimedia Appendix 3 by depressive and manic symptom severity, respectively.

**Figure 1.**
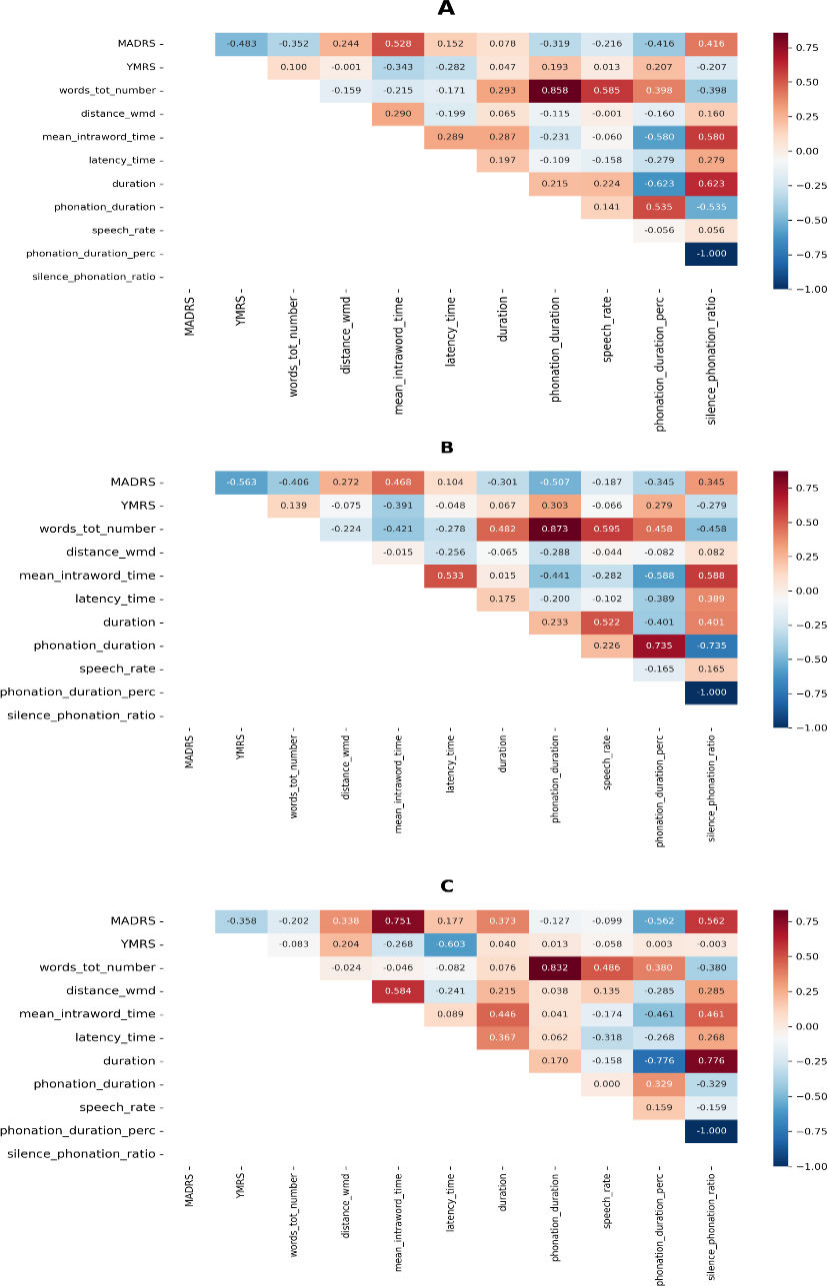
Correlation heatmap of NLP-based, semantic and conversational features in people with bipolar disorder. (A) Overall sample; (B) Male subgroup; (C) Female subgroup. MADRS: Montgomery-Åsberg Depression Rating Scale, YMRS: Young Mania Rating Scale.

In particular, grouping data into 2 categories (Multimedia Appendix 3), statistically significant differences by depressive symptoms’ severity were found for many NLP-based and conversational-like measures, including word number, phonation (also as percentage over the speech duration), and mean intraword time. Correlation analyses, based on Spearman nonparametric analysis of symptom severity continuous scores, are displayed in Figures1A-C  2A-C. These showed that both the total number of words and the length of phonation, as well as the related percentage out of segment duration, were negatively correlated (coefficients=−0.35, −0.32, and −0.42) to depressive symptoms (Figure 1A). Consistent results were observed for the ratio between silence and phonation (coefficient=0.42), as well as for mean intraword time, which was positively correlated to depressive (coefficient=0.53) and negatively to manic (coefficient=−0.34) symptoms. Among items for depressive symptoms assessment, this correlation was particularly clear between acoustic features and suicidal thoughts (coefficients ranging from 0.18 to 0.51). In addition, latency time also showed a moderate, though obviously opposite, correlation with manic and depressive symptoms, respectively (coefficients=−0.28 and 0.15).

Subgroup analyses for NLP-based and conversational features revealed more pronounced relationships in females (Figure 1C) as compared with males (Figure 1B), showing a high correlation between depressive symptoms and mean intraword time (coefficient=0.75), phonation percentage (coefficient=−0.56), and, consequently, the silence-phonation ratio (coefficient=0.56). Similarly, latency time was negatively correlated to manic symptoms among females (coefficient=−0.60).

**Figure 2.**
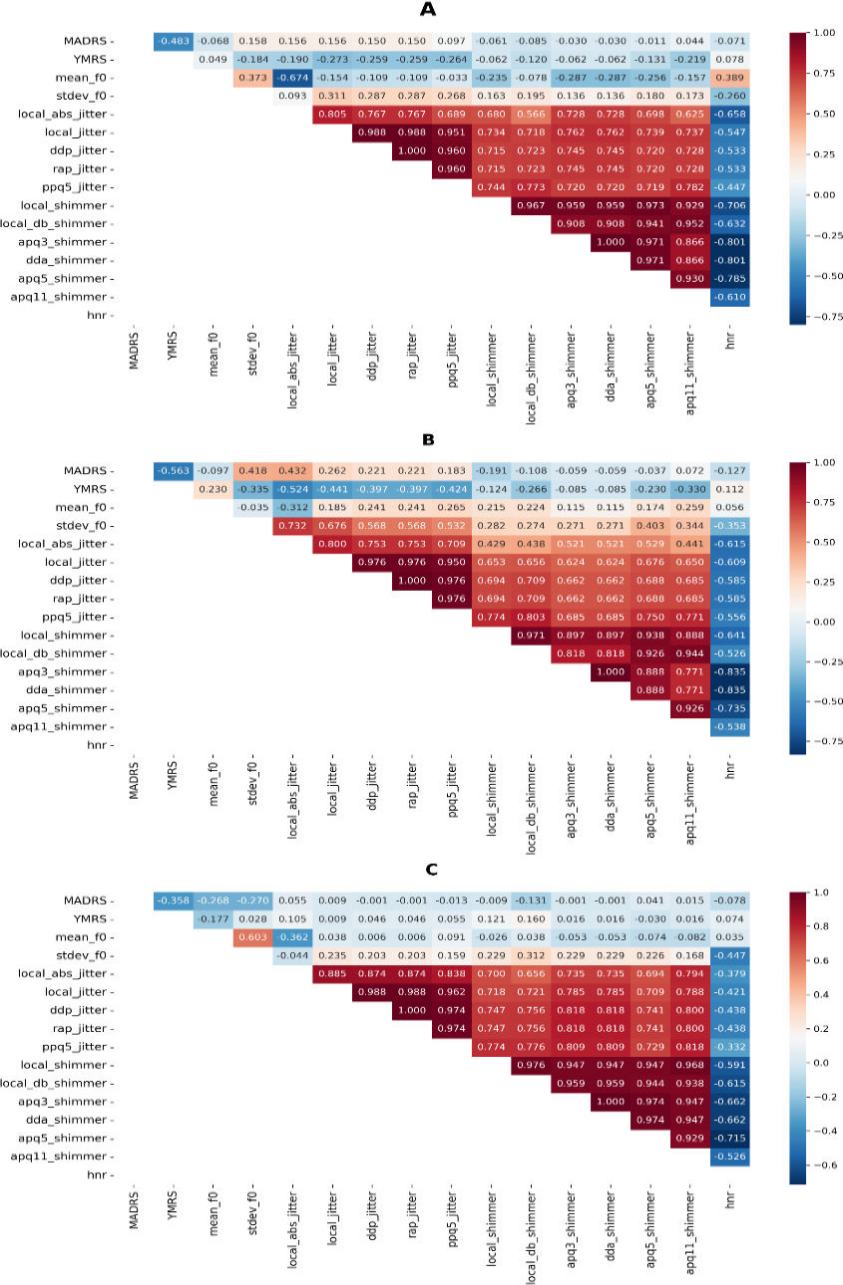
Correlation heatmap of acoustic features in people with bipolar disorder. (A) Overall sample; (B) Male subgroup; (C) Female subgroup. MADRS: Montgomery-Åsberg Depression Rating Scale, YMRS: Young Mania Rating Scale.

On the other hand, a small positive correlation was uncovered between depressive symptoms and higher values of instability in speech patterns (jitter-related indices, with coefficients ranging from 0.10 to 0.16). In contrast, small-to-moderate negative correlations were observed between manic symptoms and lower values of instability (jitter-related indices, with coefficients ranging from −0.19 to −0.27). Small estimates were found for F0, respectively (coefficient=0.16 and −0.18; Figure 2A). Except for shimmer_apq11 (manic symptoms coefficient=−0.22), we did not find any substantial relationship between shimmer-related indices (describing stable and unstable vocal intensity and speech production) and symptomatology.

Subgroup analyses suggested a role for sex also in influencing acoustic features. In particular, we found deeper connections in males as compared with females, especially in terms of F0 and jitter-related indices (Figure 2B and C).

### Predictive Models From Speech Features

Considering depressive symptoms, performance metrics showed a contribution of NLP-based and conversational features higher than what was attributable to acoustic ones (Table 2). In particular, mean intraword time, silence-phonation ratio, ppq5 jitter (ie, perturbations in F0), WMD, and percentage of phonation over duration all ranked high in terms of relative importance.

**Figure 3.**
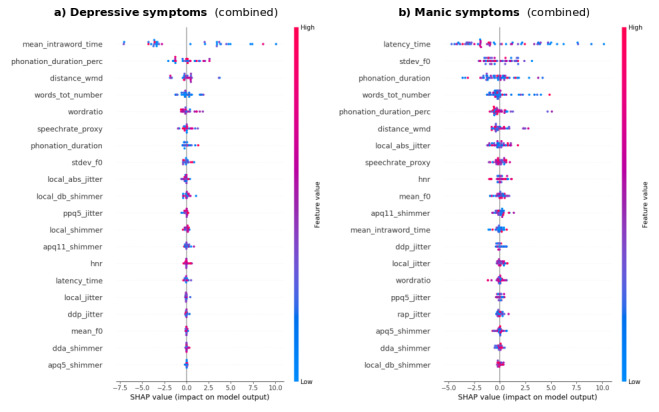
Individual features contribution to depressive and manic symptoms predictions in sex-adjusted models among people with bipolar disorder.

Including sex into the analysis, a differential contribution of various features (NLP-based and conversational vs acoustics) to the predictive models for depressive (Figure 3A) and manic (Figure 3B) symptoms can be found. However, as for manic symptoms, although a relative contribution of different NLP-based and acoustic (eg, F0 SD) features was recorded, we could not find any reliable estimates for the relevant model, even including sex. Table 2 shows detailed estimated performance metrics for testing for the trained RF regressors, even controlling for sex.

**Table 2.** Performance estimates for random forest regression models in people with bipolar disorder.

Performance^a^	Depressive symptoms		Manic symptoms	
	Unadjusted	Adjusted^b^	Unadjusted	Adjusted^b^
NLP^c^				
*R*^2^ average	0.26	0.25	—^d^	—
Fold 1	0.10	−0.55	−0.54	0.18
Fold 2	0.48	0.53	−0.13	0.02
Fold 3	0.06	0.37	0.25	0.12
Fold 4	0.54	0.64	0.23	−0.42
Fold 5	0.13	0.26	0.01	147.98
Mean squared error average	105.46	110.07	153.78	147.98
Fold 1	136.73	259.26	223.06	92.05
Fold 2	33.02	46.49	121.79	156.74
Fold 3	137.64	104.25	135.66	121.60
Fold 4	79.32	33.00	134.85	167.15
Fold 5	140.61	107.35	153.32	202.35
Mean absolute error average	8.08	8.17	10.58	10.13
Fold 1	9.58	13.64	12.47	7.79
Fold 2	3.36	5.57	9.28	10.90
Fold 3	10.31	8.56	10.40	9.26
Fold 4	7.59	4.34	9.82	9.71
Fold 5	9.26	8.74	10.96	13.00
Acoustics				
*R*^2^ average	—	0.11	—	—
Fold 1	0.29	–0.22	0.002	–0.22
Fold 2	–0.83	–0.10	–0.02	–0.15
Fold 3	–0.59	0.03	–0.14	–0.14
Fold 4	0.23	0.18	–0.38	–0.44
Fold 5	0.36	0.64	–0.28	0.01
Mean squared error average	161.64	133.75	162.86	163.51
Fold 1	47.97	222.18	68.9	125.14
Fold 2	333.00	200.40	160.47	122.80
Fold 3	202.17	85.04	185.63	175.34
Fold 4	128.54	148.52	272.25	225.23
Fold 5	96.49	12.62	127.06	170.30
Mean absolute error average	10.02	8.86	10.35	10.73
Fold 1	5.27	11.76	7.09	9.77
Fold 2	16.43	13.8	10.94	8.82
Fold 3	11.77	6.00	12.48	11.70
Fold 4	9.69	10.13	14.05	12.34
Fold 5	6.95	2.62	7.21	11.01
Combined				
*R*^2^ average	0.05	0.16	—	—
Fold 1	0.32	0.60	–0.56	–0.13
Fold 2	–0.09	0.11	0.24	0.07
Fold 3	–0.29	0.07	0.08	–0.54
Fold 4	0.10	0.04	0.06	0.18
Fold 5	0.22	0.22	–0.41	0.14
Mean squared error average	120.90	118.53	135.94	140.03
Fold 1	87.51	34.13	158.54	183.39
Fold 2	183.67	111.11	60.67	122.32
Fold 3	184.71	164.45	192.81	126.43
Fold 4	47.71	148.83	178.84	112.36
Fold 5	100.91	134.10	88.83	155.68
Mean absolute error average	8.65	8.68	9.61	10.00
Fold 5	6.69	4.37	11.21	11.50
Fold 5	11.49	7.33	6.95	8.86
Fold 5	11.04	11.29	11.40	10.30
Fold 5	5.57	10.15	11.19	8.26
Fold 5	8.46	10.26	7.28	11.08

a Metrics for testing based on a nested cross-validation approach (pilot, cross-sectional study, N=32). Range for symptom scores: 0‐60.

b Including sex.

c NLP: natural language processing.

d Not available.

## Discussion

### Main Findings

This study aimed at piloting the simultaneous use of speech acoustics, as well as natural language features, to glean insights into BD depressive and manic symptoms. Our findings corroborate evidence on the relationships between symptom severity and speech features, supporting the potential predictive role for clinical purposes of digital mental health applications, embedded in a mHealth integrated system.

First, the speech of participants with BD showed that vocal perturbations (eg, higher instability and hesitations considering voice quality), latency time, and increased silences and pauses over time speaking all correlated to depressive symptoms. Consistently, increased depressive symptoms resulted in NLP-based features such as a smaller number of words and longer mean intraword time, with lower pressure of speech. In our exploratory study, this relationship was particularly clear among females. This effect was corroborated by the predictive model, showing a contribution of NLP-based and conversational features higher than for acoustic ones. This finding aligns with prior evidence, advocating that text-based features contribute more to model accuracy than audio parameters [18]. However, also the latter component (ie, fundamental frequency, jitter- and shimmer-related indices) deserves a careful assessment, since our findings show that these indices might have an impact at least among males to predict future episodes. Indeed, recent evidence from healthy populations sheds light on sex differences in speech markers (eg, prosodic features) with different acoustic cues conveying various emotions [50]. A combination of inherent biological dissimilarities, socialization processes, influences of the social environment, and cultural expectations might contribute to these differences in both expression and perception of related emotional prosody [52,53]. Moreover, individuals may modulate their speech to align with the dominant pitch range within a specific linguistic community [54], and similar modulation may occur in conversational dialogues versus monologues and in spontaneous versus elicited speech. Thus, this criterion should be taken into account when designing apps with speech recognition and processing tasks for people with BD [31].

Second, voice instability and hesitations, as well as mean intraword time, were negatively correlated to manic symptoms. However, the interpretation of the relationship between manic features and vocal abnormalities is not straightforward. Mixed findings emerged on the relationships between speech features and manic symptoms, preventing us from supporting our original hypothesis. One plausible explanation may stem from the sample characteristics. Indeed, our participants were more likely to report depressive symptoms, and just a few had severe manic features.

However, the overall moderate correlations between speech markers and symptom severity were consistent with previous work that used speech smartphone data to discriminate between different mood states [20,21]. It has been argued that speech features may be useful to detect a trait [55] rather than a state [56] in BD. However, alterations in voice perturbations have been observed when assessing vocal markers of suicidal ideation [57], and this makes further research for vocal features reasonable, at least for depressive conditions.

### Smartphone-Based Applications

Consistent with previous research on smartphone-based applications designed to record and analyze speech patterns in real time, our findings emphasize the feasibility of a simple, yet clinically useful, application of digital technology [13]. In particular, we developed the frontend of the app as a basic digital environment, freely managed by participants on their own smartphones. Participants reported a high level of engagement with the tool, showing an acceptable system usability level as assessed by SUS [35], without perceiving intrusiveness of the recording of both elicited and spontaneous conversations.

Comparisons of the vocal performance of people with BD with unaffected relatives and healthy controls have shown a clear speech “fingerprint” of the clinical condition [58], suggesting the utility of multilevel inputs [59]. However, there is also the need for a wider understanding of fluctuations in symptom severity and mood states in this population [60]. The major strength of our study consists in the usefulness of different speech data (eg, linguistic, conversational, acoustics) to differentially identify symptoms of BD. Thus, for relapse prevention purposes, future research should possibly explore systems combining smartphone-based generated objective acoustics data with additional information, such as from facial expressions and gestures [61]. This would ultimately improve BD state prediction, even considering classification tasks [21,62-64].

### Clinical Implications: Interdisciplinary Perspective

This pilot study represents a step forward in the identification and utilization of digital biomarkers for BD from natural language and audio streams, with implications for personalized mental health care and early intervention strategies. Our approach holds promise for complementary, remote assessments enhancing depressive and partly manic states prediction by exploiting participants’ speech. This would have significant implications, especially considering BD fluctuating symptomatology. Nonetheless, leveraging live speech recordings as a predictive tool, repeated assessments are needed to identify individuals at risk of transitioning to depressive and manic states.

Despite promising findings from automated assessments, mental health care heavily relies on participant interviews, yet with often subjective reports, cognitive limitations, and stigma [18]. Integrated systems, aiming at taking advantage of candidate digital markers from speech recognition, would possibly boost a care approach in which digital technology enhances, but does not replace, existing models from clinical assessment [30]. Indeed, automated assessment does not inherently lead to adherence and engagement of individuals with BD [65].

Finally, clinical, hypothesis-driven research on BD should not be dismissed, since algorithms may not be considered a black-box replacement for traditional data modeling, but they rather integrate with other systems, embedding a substantial clinical validation [66,67].

### Limitations and Future Directions

We should acknowledge some limitations of this study. Analyzing speech and natural language in individuals with BD implies a challenge due to the nature of the disorder and to ethical considerations.

First, properties of chosen machine-learning models may hamper identification of unknown patterns based on values that fall outside the training set. Effective NLP and supervised learning models may require high-quality, annotated datasets. While exploratory in nature, the study’s limited sample size may have constrained the model’s statistical power and the ability to capture the full complexity of the underlying data distribution, thereby hindering meaningful subgroup comparisons. Our preliminary findings should be replicated and extended in a larger, more diverse sample of people with BD to mitigate the risks associated with overfitting. Furthermore, future research should address classification approaches based on severity thresholds for both MADRS and YMRS. Accordingly, there is potential for alternative modeling approaches for regression tasks (eg, splines) that might be implemented in the future. While still considering the number of predictors, these may possibly enable a better understanding of the nature of the existing relationships and nonlinear patterns.

Consistently, the lack of standardized (linguistic and acoustic) markers represents a barrier when studying relationships with mood states. Indeed, the model may still learn to overfit to irrelevant or noisy features the data may contain, especially if they are informative in the training set by chance.

Furthermore, the speaker’s identity may show a possible confounding role in a between-subject design. Therefore, studies with a longitudinal design (ie, within-subjects) should be recommended, deploying Ecological Momentary Assessment approaches [24,68]. In addition, speech patterns may generate misinterpretations if individual cultural and linguistic factors are not accounted for [69]. Similarly, speech during manic episodes may exhibit circumstantiality or tangentiality, where individuals provide excessive details or veer off-topic. Rapid speech, tangential thinking, or unconventional language use pose challenges for automatic speech recognition systems. Analyzing such complex speech patterns requires a deep evaluation of language and context, achieving appropriate understanding of an individual’s usual way of communicating in order to distinguish changes associated with BD episodes.

Furthermore, in our study, speech features were averaged over relevant duration, thus constraining the role of temporal variations across related measures in predicting symptom severity. Future research should endeavor to integrate dynamic aspects of speech on mood states transitioning.

Finally, other clinical variables, not investigated in our sample, are likely to influence the individual’s speech. For instance, it should be noted that anxiety and anxious distress, often co-occurring with bipolar depression [70], may significantly influence speech features [71], as well as medication prescribed [72-74] and drug or alcohol comorbid conditions [75].

### Conclusions

Speech patterns, underlying both linguistic and acoustic features, are able to yield quantifiable differences, thus embodying digital markers of symptom severity. Multimodal, smartphone-integrated digital assessments could serve as powerful tools for clinical purposes to remotely complement standard mental health evaluations, potentially contributing to distinguish clinical conditions in people with BD. Feasibility of similar systems seems promising, though issues related to privacy, intrusiveness, and clinical therapeutic relationships should be carefully considered.

## Supplementary material

10.2196/65555 Multimedia Appendix 1 Checklist.

10.2196/65555 Multimedia Appendix 2 Features.

10.2196/65555 Multimedia Appendix 3 Supplementary analyses.

## Abbreviations

BD: bipolar disorder
DSM-5: Diagnostic and Statistical Manual of Mental Disorders, Fifth Edition
MADRS: Montgomery-Åsberg Depression Rating Scale
MAE: mean absolute error
mHealth: mobile health
MSE: mean squared error
NLP: natural language processing
RF: random forest
SCID-5: Structured Clinical Interview for DSM-5
STROBE: Strengthening the Reporting of Observational Studies in Epidemiology
WMD: word mover’s distance
YMRS: Young Mania Rating Scale
