# Supplementary material for: Acoustic and Natural Language Markers for Bipolar Disorder: A Pilot, mHealth Cross-Sectional Study
Source: JMIR Form Res. 2025 Apr 16;9:e65555. doi: 10.2196/65555 (PMC12017610; doi:10.2196/65555)
Supplement: Multimedia Appendix 2 [file formative-v9-e65555-s002.docx]

**Appendix 2.**

**Table 1.** NLP-based, semantic, conversational, and acoustic features from speech

| ***Features (Type)*** | ***Description*** | ***Remarks*** |
| --- | --- | --- |
| *NLP-based and Semantic* | | |
| Words total number | Actual total number of words produced: NLP-based score showing the total number of words articulated by participant during speech | number |
| Word Mover’s Distance | Measure of semantic similarity between target text and recalled text, capturing both lexical overlap and semantic similarity. | *-* |
| Mean intra-word time | Mean silence time between produced words, referring to the average time taken to articulate or pronounce subsequent words | msec |
| Word ratio | number of words produced that find match in the story text out of total number of words | - |
| *Conversational* | | |
| Latency time | Silence time from recording onset to first voiced production. It indicates the response time when starting the task, i.e., the time taken to produce the speech sample (Wang, 2008) | msec |
| Speech duration | Length of recording | sec |
| Phonation | Time speaking within speech duration.  It embodies total time of voiced recording identified by the presence of pitch in recording by PRAAT functions. | sec |
| Speech rate | Calculated as the number of words within a defined time interval is a proxy for Speech rate. It was based on individual speech duration (i.e., phonation time, sample median: 45.1 - iqr: 85.62 sec) | words per sec |
| Phonation/duration | total time spent speaking divided by speech sample duration, showing the amount of voiced recording expressed as percentage | % |
| silence | Total time of silent recording, including pauses during speech (e.g., intraword time) | sec |
| silence/phonation | Ratio between pauses during speech and time speaking (i.e., phonation) | - |
| *Acoustics* | | |
| Fundamental frequency (F0) | lowest frequency of a periodic waveform, conveying linguistic information simultaneously with paralinguistic (e.g., emotion, emphasis) information. It depends on the number of vocal fold vibrations producing sound waves during a given time period. The more quickly the vocal folds vibrate, the higher the F0. During unvoiced speech, F0 = 0.  F0 varies with individual anatomy, vocal folds state and position, and airflow conditions. Although there is a typical range of values for the different genders and ages (the F0 baseline for modal phonation is set at 70 Hz and the F0 topline is set at 175 Hz for males and 275 Hz for females (Johnson K. Language Arts & Disciplines - Acoustic and Auditory Phonetics, 2003, Wiley), related measure is not stationary since F0 may depend on factors such as the state of mind of the person, the time of day that fit the lifestyle and professional use of voice. | Hertz (Hz)  *mean* and *sd* to minimize speaker dependence |
| Jitter local_abs | the average absolute difference between consecutive intervals | *%* |
| Jitter local | the average absolute difference between two consecutive intervals, divided by the average interval | *%* |
| Jitter ddp | Difference of Differences of Periods, representing the average  absolute difference between the differences between consecutive intervals, divided by the average interval. | *%* |
| Jitter rap | Relative Average Perturbation: the average absolute difference of one interval and the average of the period with its two neighbours, divided by the average interval | *%* |
| Jitter ppq5 | five-point Period Perturbation Quotient: the average absolute difference between an interval and the average containing its four nearest neighbour intervals, i.e. two previous and two subsequent intervals, divided by average interval. | *%* |
| Shimmer local | It represents the average absolute difference between the amplitudes of two consecutive periods, divided by the average amplitude.  Higher values indicate greater variability or instability, while lower ones suggest more consistent and stable vocal intensity (i.e., smoother and more regular speech production). | % |
| *Shimmer local_db* | Represents the average absolute logarithm of the difference between the amplitudes of two consecutive periods | *Decibel (dB)* |
| Shimmer apq3 | Three-point Amplitude Perturbation Quotient, representing the average absolute difference between the amplitude of a period and the mean amplitudes of its two neighbours, divided by the average amplitude. | *Decibel (dB)* |
| Shimmer dda | Average absolute difference between consecutive differences among the amplitudes of consecutive periods (3*shimmer apq3) | *Decibel (dB)* |
| Shimmer apq5 | Five-point Amplitude Perturbation Quotient, representing the average absolute difference between the amplitude of a period and the mean amplitudes of it and its four nearest neighbours, divided by the average amplitude | *Decibel (dB)* |
| Shimmer apq11 | Eleven-point Amplitude Perturbation Quotient, representing the average absolute difference between the amplitude of a period and its ten closest neighbours, divided by the average amplitude. | *Decibel (dB)* |
| Harmonic-to-Noise ratio (HNR) | Voice quality, computed as the ratio between periodic and non-periodic components of a voiced sound.  Higher values indicate harmonic voice. Conversely, lower HNR values indicate underlying vocal cord tension or irritation, thus possibly suggesting emotional distress. | *Decibel (dB)* |
