# Supplementary material for: Acoustic and Natural Language Markers for Bipolar Disorder: A Pilot, mHealth Cross-Sectional Study
Source: JMIR Form Res. 2025 Apr 16;9:e65555. doi: 10.2196/65555 (PMC12017610; doi:10.2196/65555)

**Appendix 3**

**Figure S1a**. Features from participant’s speech by depressive symptoms (severe vs. non-severe symptoms)


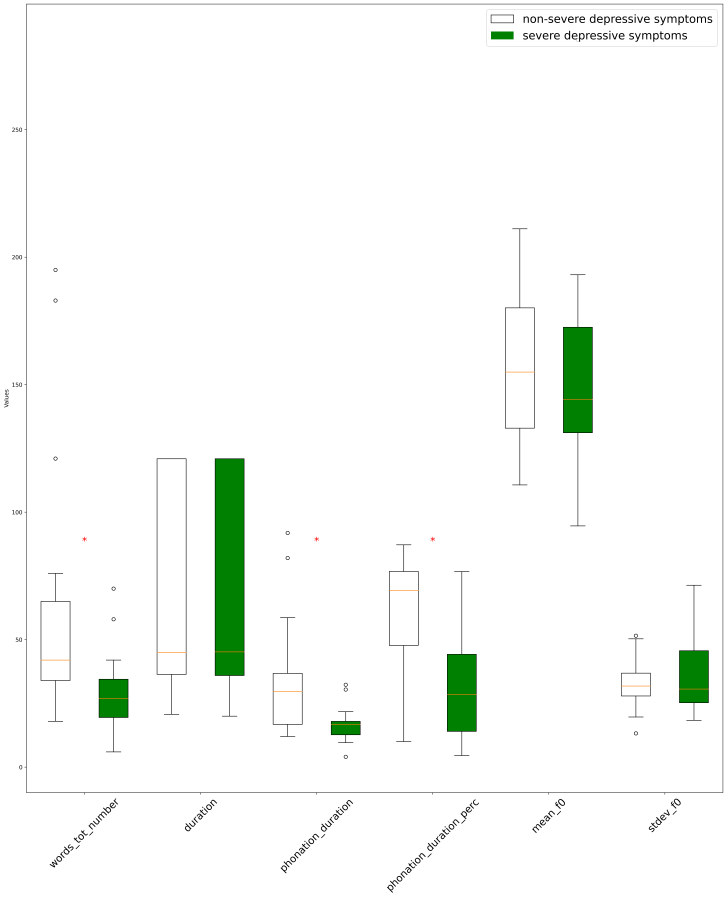


**Figure S1b**. Features from participant’s speech by depressive symptoms (severe vs. non-severe symptoms)


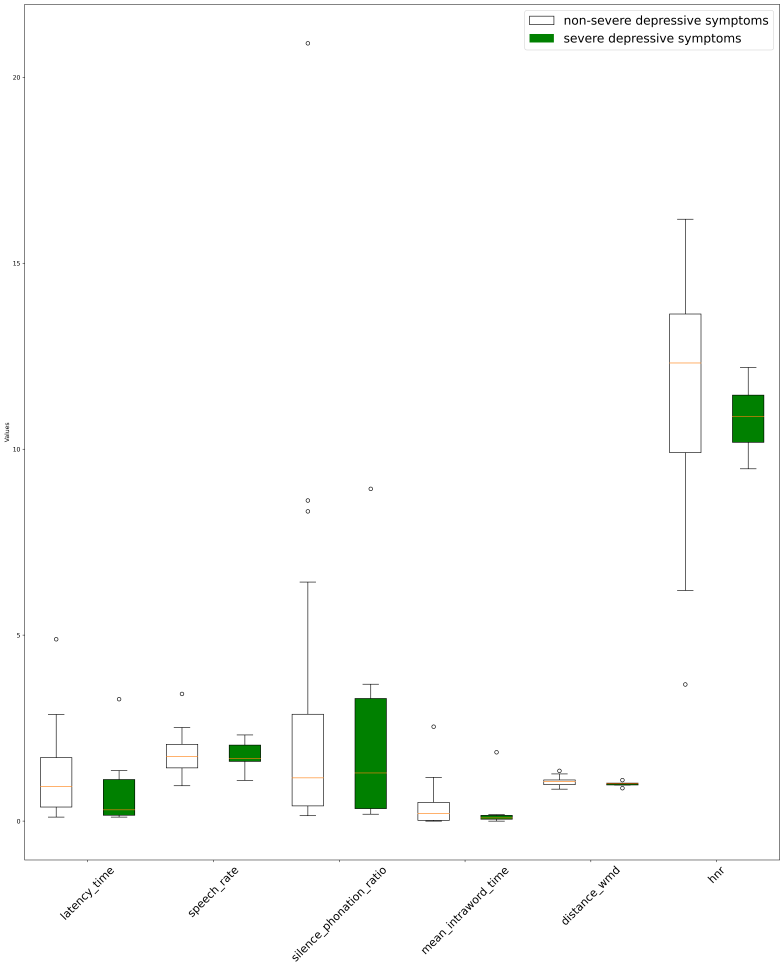


**Figure S1c**. Features from participant’s speech by depressive symptoms (severe vs. non-severe symptoms)


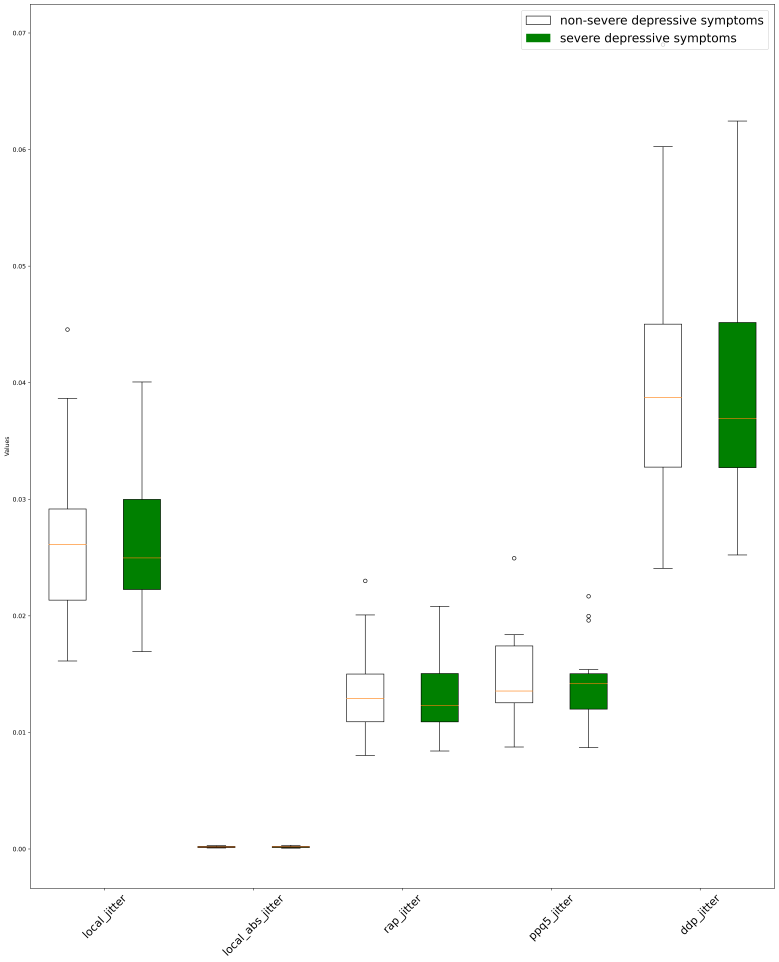


**Figure S1d**. Features from participant’s speech by depressive symptoms (severe vs. non-severe symptoms)


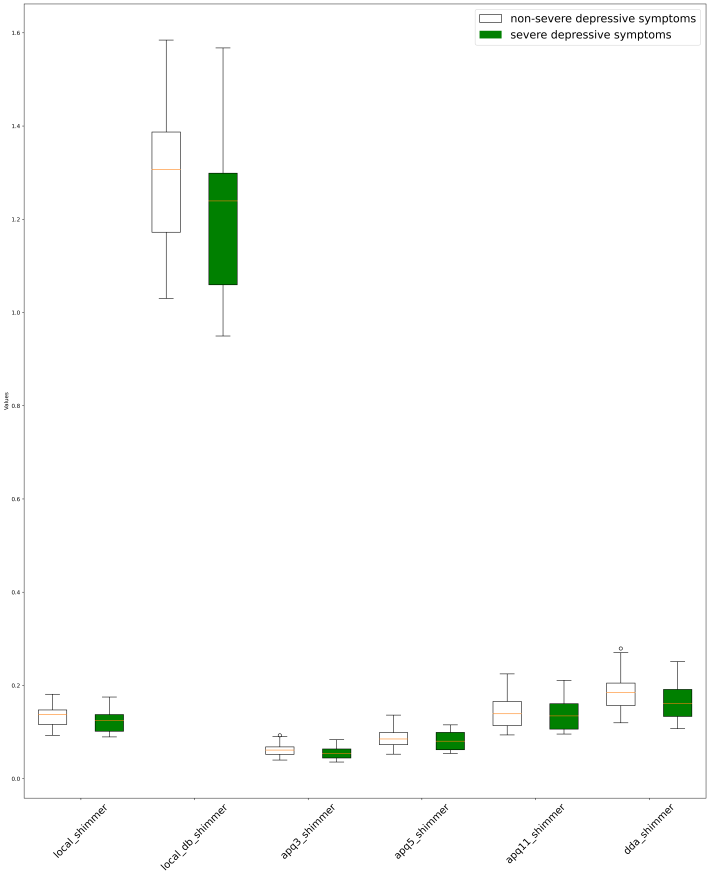


**Figure S2a**. Features from participant’s speech by manic symptoms (severe vs. non-severe symptoms)


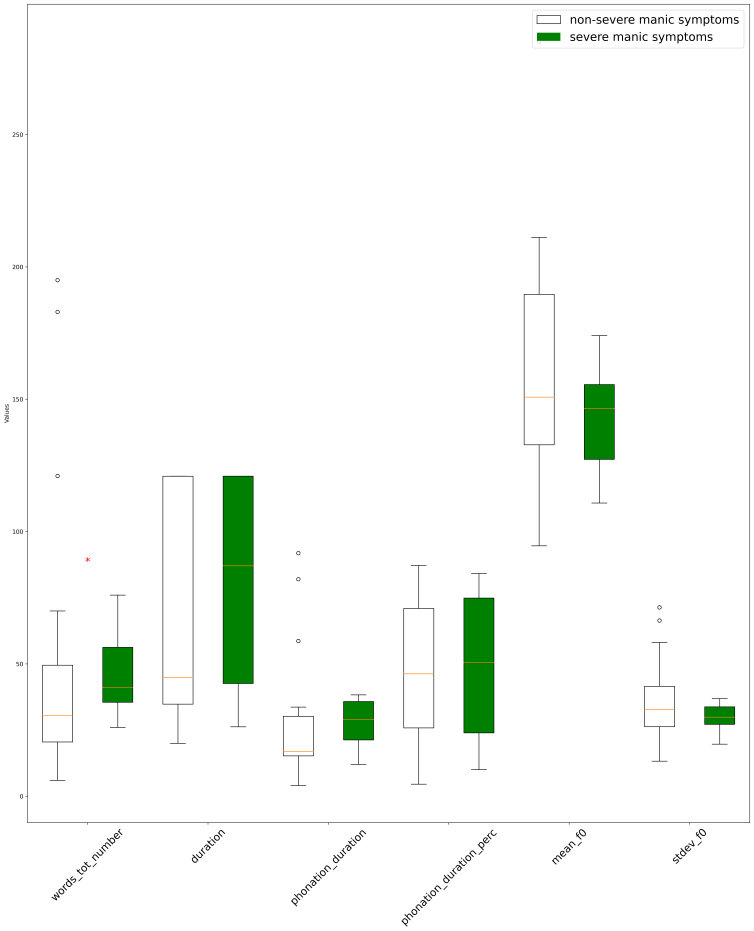


**Figure S2b**. Features from participant’s speech by manic symptoms (severe vs. non-severe symptoms)


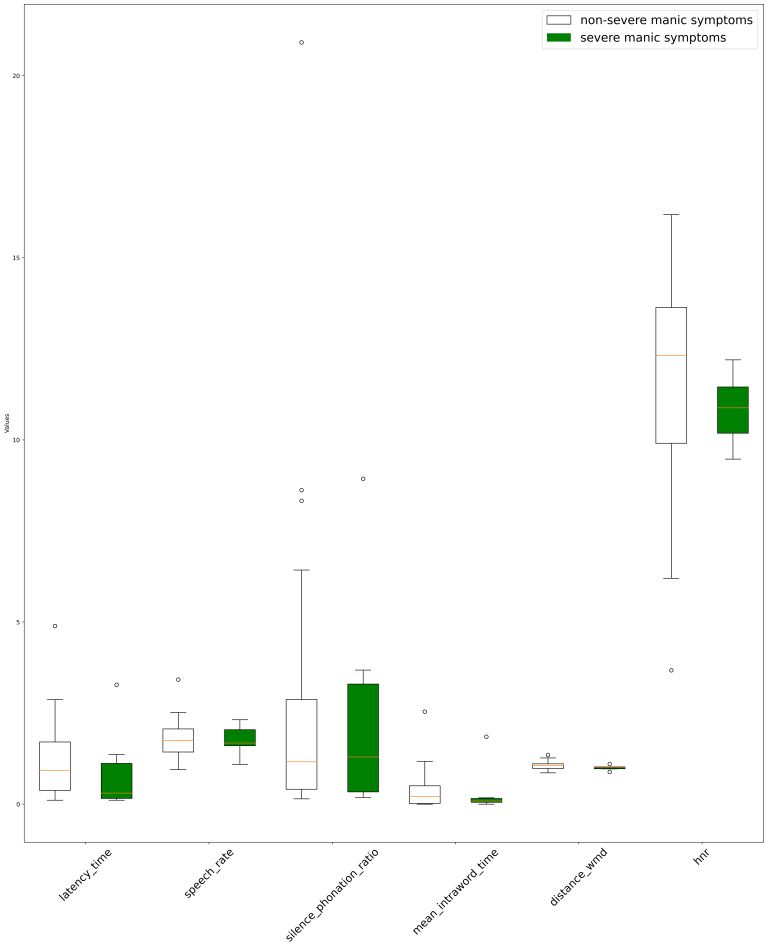


**Figure S2c**. Features from participant’s speech by manic symptoms (severe vs. non-severe symptoms)


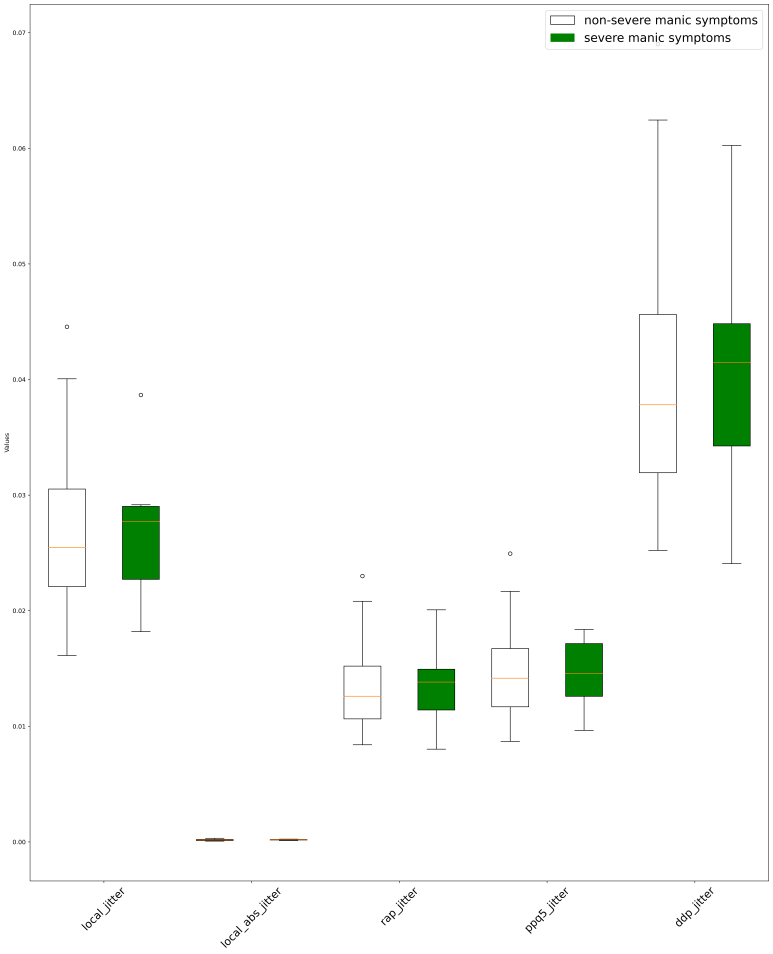


**Figure S2d**. Features from participant’s speech by manic symptoms (severe vs. non-severe symptoms)


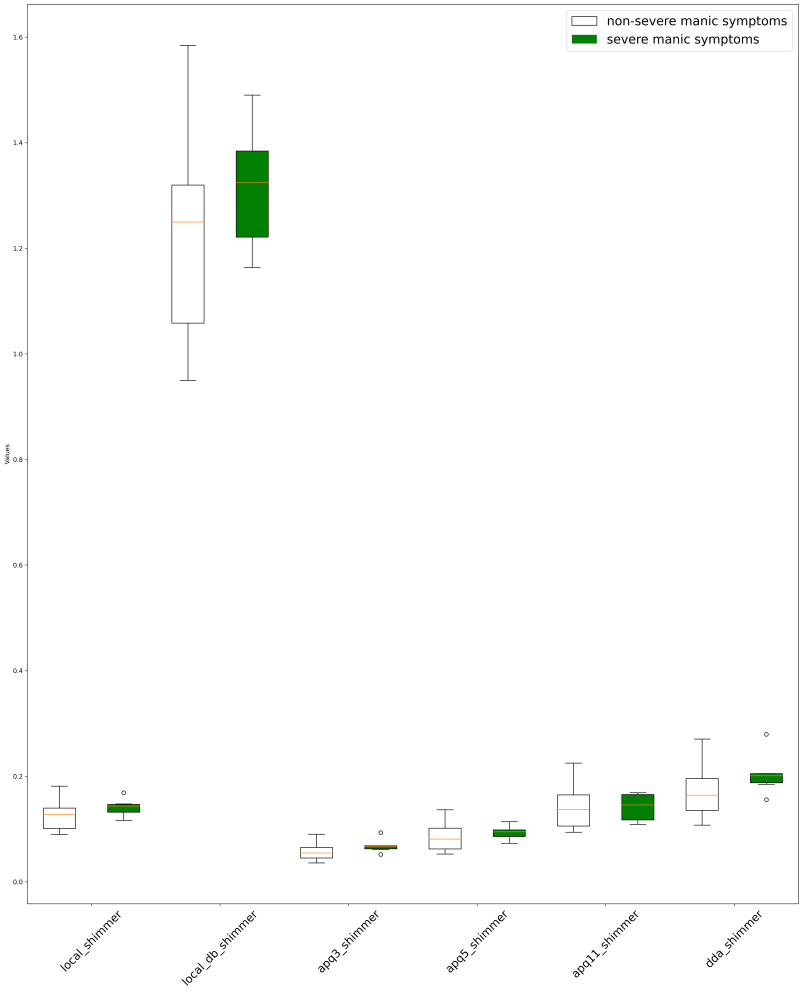

Supplement: Multimedia Appendix 3 [file formative-v9-e65555-s003.docx]
